# Supplementary material for: Co-design of Lifestyle6, a digital tool targeting multiple health behaviour changes for cancer risk reduction and early detection support
Source: PLoS One. 2026 Apr 16;21(4):e0347311. doi: 10.1371/journal.pone.0347311 (PMC13086309; doi:10.1371/journal.pone.0347311)
Supplement: S2 File — (DOCX) [file pone.0347311.s002.docx]

**S2 File. Project team.**

Four trained female facilitators experienced in cancer research and qualitative methods hosted the community panel workshops. All other administrative tasks (i.e., communication with panel members, scheduling of workshops, reimbursement, etc.) were handled by the project lead (BM), who brought substantive expertise in the field and a vested interest in developing a successful programme that could be implemented and evaluated. BM is a postdoctoral researcher with a background in behavioural science, chronic disease prevention and implementation research, experienced in digital health and consumer engagement. Additional facilitation was provided by research assistants (BV, KC and EP) from the existing research team, who were selected based on their relevant backgrounds in, methodological expertise and availability to support the co-design process. BV is a postdoctoral researcher with a background in nursing and public health, experienced in early detection, cancer treatment and survivorship support and qualitative research methods. KC is a PhD candidate with a background in health psychology, bowel cancer screening participation, experienced in qualitative research methods. EP is a research assistant with a Masters of Epidemiology and a background in health sciences, experienced in consumer engagement and qualitative research methods. BM has previously undertaken extensive co-design training and used established frameworks and recommendations to develop workshop content and activities.

The Lifestyle 6 prototype was designed and developed by HG, a lead education and information specialist at Cancer Council Queensland. HG has a background in psychological sciences, human-centered design, instructional design, and eLearning development, and is experienced in transforming complex challenges and ideas into impactful, user-focused solutions. HG did not attend any of the group-based panel workshops but received a comprehensive summary of insights from those workshops and was briefed on the key project aims prior to designing the prototype.
