# Supplementary material for: Co-design of Lifestyle6, a digital tool targeting multiple health behaviour changes for cancer risk reduction and early detection support
Source: PLoS One. 2026 Apr 16;21(4):e0347311. doi: 10.1371/journal.pone.0347311 (PMC13086309; doi:10.1371/journal.pone.0347311)
Supplement: S3 File — (PDF) [file pone.0347311.s003.pdf]

Expression of Interest Form

COMMUNITY ENGAGEMENT OPPORTUNITY

EXPRESSION OF INTEREST FORM FOR COMMUNITY MEMBERS

Why we need your views.

Many cancers can be prevented. Health and lifestyle choices, such as physical activity, diet, sun protection, tobacco use and alcohol consumption are known to affect a person's risk of developing cancer. In addition, regular cancer screening greatly improves the chances of early detection, which leads to better outcomes following a diagnosis.

To reduce the number of new cancer cases and to save lives, it is important to encourage the wider community to prioritise healthy lifestyle behaviours and to participate in existing initiatives for the early detection of cancer. We want to improve how we design, deliver and promote community-wide cancer prevention and screening programs for maximal impact. So we would like to know how people prefer to receive digital health information and what may help them use this information effectively to reduce their cancer risk and participate in screening programs.

For this project, we are looking to form an online panel of community members who will help us develop a digital cancer prevention and screening research platform (i.e., website or application) to faciliate ongoing community-led cancer prevention research and to design and distribute freely accessible tools and resources.

What you need to do.

As a panel member, we ask you to join 3 separate online meetings (i.e., video conferencing) to take part in virtual group discussions that may include brainstorming ideas and providing feedback on digital design.

Meetings will take place over the next 6-12 months. Each meeting will have a duration of 1-2 hours. Meetings will be scheduled at a suitable time on weekdays (Mon-Fri). In addition, we ask you to complete an individual online activity where you will test the new platform once it has been developed. This will take approximately 30-60 minutes to complete. At the end, you will be invited to answer a short online survey that takes approximately 20-30 minutes to complete.

As a panel member, you will be reimbursed with a grocery voucher (i.e., Coles e-gift card) valued at $50 per hour of your time for each meeting you attend and online activity that you complete, as a thank you for your contribution.

EXPRESSION OF INTEREST FORM FOR COMMUNITY MEMBERS

Who we are looking for.

At Cancer Council Queensland, we listen to, work with and support people from all types of backgrounds. For this particular project, we would like to hear from Australian adults with an interest in cancer prevention.

Why we need your personal details.

Please read this information carefully before you proceed to complete an expression of interest form.

The personal information you are providing to us is collected for the purpose of helping us match you to different panel activities we have planned for this project. By expressing your interest in participating in these panel activities, you agree to us collecting that information for the sole purpose described above. Your data will be stored securely on our database and accessed only by research team members.

Please note: This form does not guarantee a position on the panel. As places are limited, the information you provide in this form will assist us in making sure we consider people from a variety of backgrounds. Panel members will be selected at the end of the month and you will be notified of the outcome via email and/or phone.

If you have any questions about this form or the project, please don't hesitate to contact the research team:

Larry Myers:

LarryMyers@cancerqld.org.au

+61 7 3634 5120

Beatrice Murawski:

BeatriceMurawski@cancerqld.org.au

+61 7 3634 5251

You may view and/or download our Terms & Conditions before you proceed.

[Attachment: "Terms and Conditions.pdf"]

PARTICIPANT VERIFICATION

We ask you to answer the following questions truthfully to be considered as a participant in this project. The first question will help us to verify that you are a real person filling in this form.

Please select the following 4 letters from the list:<br><br>P, R, I, O	☐ P<br>☐ R<br>☐ E<br>☐ V<br>☐ E<br>☐ N<br>☐ T<br>☐ I<br>☐ O<br>☐ N
Please enter today's date by clicking on the Today button.	________________________

PARTICIPANT ELIGIBILITY

Do you live in Australia?	◯ Yes<br>◯ No
Are you at least 18 years of age?	◯ Yes<br>◯ No
Are you able to read, speak and write in English language?	◯ Yes<br>◯ No
Do you have permanent access to the internet on a device that supports video calls?	◯ Yes<br>◯ No
Will you be able to attend online panel meetings on three separate occasions (with a duration of 1-2h each) over the next 6-12 months?	◯ Yes<br>◯ No

PARTICIPANT DETAILS

Thank you for confirming your eligibility.

We collect the following personal information to be able to match you to different panel activities we have planned for this project and to contact you to inform you about the outcome of your expression of interest. Your information will not be used for any other purpose.

What is your name?

Please enter your full name, for example: John Smith

____

What is your year of birth?

Please enter your birth year without the day or month, for example: 1986

____

What is your gender?

Please select/specify the gender you identify with.

◯ Male
◯ Female
◯ Other

Please specify what gender you identify with

____

What is your postcode?

Please enter your 4-digit postcode, for example: 4005

____

Were you born in Australia?

◯ Yes
◯ No

Which country were you born in?

____

What is your occupation or area of work/study?

For example: Early Childhood Education and Care.

____

REASONS FOR PARTICIPATION

What are your main reasons for participating in this project?

An example may be that you or someone close to you has been affected by cancer, or you are interested in making a difference to cancer prevention research, or you feel curious about the work a community panel does. (Please note, there are no right or wrong answers to this question).

____

What time slots would suit you best to participate in our online activities? (You may select multiple options)

	Not available	9am - 11am	11am - 1pm	1pm - 3pm	3pm - 5pm	5pm - 7pm
Mondays	☐	☐	☐	☐	☐	☐
Tuesdays	☐	☐	☐	☐	☐	☐
Wednesdays	☐	☐	☐	☐	☐	☐
Thursdays	☐	☐	☐	☐	☐	☐
Fridays	☐	☐	☐	☐	☐	☐

CONTACT DETAILS

To inform you of the outcome of this expression of interest, we need to be able to contact you.<br><br>Please tell us how you would like to be contacted.	☐ Email<br>☐ Call/SMS<br>☐ Both
Please enter your email address	__________________________________
Please enter your phone number using numbers only	__________________________________

END OF FORM

Thank you for completing this form.

Please note, submitting an expression of interest form does not constitute membership or a place on the community panel that will be consulted for this project. We will contact you as soon as places have been allocated.

Please contact us if you have any questions about this.

Larry Myers:

LarryMyers@cancerqld.org.au

+61 7 3634 5120

Beatrice Murawski

BeatriceMurawski@cancerqld.org.au

+61 7 3634 5251
