# Supplementary material for: Co-design of Lifestyle6, a digital tool targeting multiple health behaviour changes for cancer risk reduction and early detection support
Source: PLoS One. 2026 Apr 16;21(4):e0347311. doi: 10.1371/journal.pone.0347311 (PMC13086309; doi:10.1371/journal.pone.0347311)
Supplement: S4 File — (DOCX) [file pone.0347311.s004.docx]

**
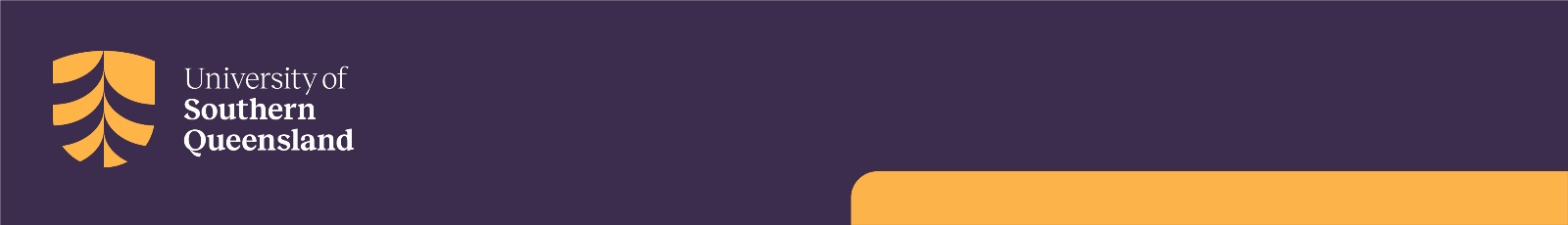
**

**UniSQ HREC Approval number: ETH2023-0359**

University of Southern Queensland

Participant Information Sheet

Consumer Advisory Groups

| **Project Title** |
| --- |
| Consumer Consultation for Cancer Prevention Research |

| **Research team contact details** | |
| --- | --- |
| **Principal Investigator Details** | **Co-Investigator Details** |
| Dr Larry Myers  Email: LarryMyers@cancerqld.org.au  Telephone: +61 7 3634 5120 | Dr Beatrice Murawski  Email: BeatriceMurawski@cancerqld.org.au  Telephone: +61 7 3634 5251 |

| **Description** |
| --- |

The purpose of this project is to learn about your views of digital health information related to cancer prevention and better understand your needs and preferences when accessing this information. Having this insight will help us develop a new and freely accessible digital platform with evidence-based resources and strategies that help Australian adults make changes to their health and lifestyle behaviours to reduce their cancer risk.

Before we make this platform available to the wider community, you will be invited to test it, which is a way for us to ensure your thoughts and views have been considered and the platform is user-friendly, appropriate, and relevant. In addition, we would like to know about your experience as a consumer panel member and this will help us decide how we run these meetings in the future.

| **Participation** |
| --- |

**Consumer Panel Meetings**

Your participation will involve contributing your thoughts and ideas in a virtual group setting (i.e., using online video conferencing) on three separate occasions. Each meeting will have a duration of approximately 1 to 2 hours. There will be a total of 7 to 10 members per group and each panel meeting will be guided by a trained facilitator, who is a member of our research group. You will participate in open discussions, brainstorming and mapping activities.

All online panel meetings will take place between late 2023 and late 2024. We will send you a link and instructions on how to join the meeting and further information regarding the ground rules of participating and the goals of each meeting. You will be able to ask questions at any time before, during or after the meeting. You will also have the option to schedule a one-on-one phone call with a member of the research team if you feel that you were unable to raise anything you wanted to contribute during the group sessions, or you have any other concerns regarding your participation.

The first meeting will help us become familiar with your views of digital health information related to cancer prevention and better understand your needs and preferences and potential barriers to accessing this information. In the second meeting, you will be able to help us explore potential solutions based on the needs and preferences that we have identified in the first meeting. We will then take this information to our team of web developers and information technology experts, who will build a prototype (i.e., test version) of the platform. In the third meeting, we will present prototypes to you (and the group) and you will be able to share with us what you like and don’t like about the prototypes and why you like or dislike certain aspects of them. We will then take these insights back to our developer team to finalise the prototype.

Approximately 3 months after the third meeting, you will receive a link to the final version of the platform and instructions on how to access and use it. We will ask you to think aloud while testing and browsing this version and submit a recording of your thoughts. This will take approximately 30–60 minutes of your time and will be an individual activity with no other group members or facilitators present, so you can complete this at a time that suits you best.

**Online Questionnaires**

Following the ‘think aloud’ activity (i.e., testing the platform and recording your impressions) described above, you will be directed to a short online questionnaire where we will ask you to rate your experience as a consumer panel member. This questionnaire will take approximately 15 to 30 minutes of your time.

The questionnaire will ask you to what extent you feel that your voice was heard and understood during the panel meetings, and if you think your contributions had an influence on the development of the prototype and whether you felt comfortable being a member of the consumer group.

**Your Participation**

Your participation in this project is entirely voluntary. If you do not wish to take part, you are not obliged to. If you decide to take part and later change your mind, you are free to withdraw from the project at any stage without penalty.

Due to the collaborative nature of the group-based panel meetings and each meeting being recorded, we will be unable to delete any information you contributed from our recordings once you have participated in the meeting/s. However, if you notify us that you wish to withdraw parts of or all your data, we can exclude your data from any subsequent evaluation and publication. You are not required to give reasons for such a request.

Any data you have provided during your individual testing and recording activity and in the online questionnaire may be withdrawn and confidentially destroyed without you having to give reasons for requesting this. However, you will be unable to withdraw data collected about yourself after the data has been analysed and/or published. We will only publish group level data which does not identify you as an individual.

If you do wish to withdraw from this project or withdraw data collected about yourself, please contact the Research Team (contact details at the top of this form). Your decision whether you take part, do not take part, or take part and then withdraw, will in no way impact your current or future relationship with the University of Southern Queensland and/or Cancer Council Queensland.

| **Expected benefits** |
| --- |

It is expected that your participation in this project will have direct and indirect benefits for you. These include:

- Increased sense of ownership and empowerment to use health information related to cancer prevention and share your knowledge with your network of family and friends.
- Greater knowledge of scientific research processes.
- A deeper understanding of cancer prevention strategies, risk factors and behavioural modifications.
- Increased intention to make positive changes to a range of health behaviours including physical activity, diet, tobacco use, alcohol consumption, sun protection and early detection (i.e., screening) routines.
- Improvements in a range of health outcomes (e.g., cardiovascular health, blood glucose control, weight management, mood and quality of life, productivity, and overall mortality risk) as a result of changes in one or several health behaviours.

As a thank you for your time, you will receive a gift card (i.e., grocery voucher) to the value of $50 per hour for each panel meeting attendance and card for completing the individual think aloud activity and subsequent online survey. If you attend all 4 activities, you will receive a total of 4 gift cards [with an approximate value of $200]. You are free to decline this reimbursement if you wish to do so by letting the research team know. We intend to send each gift voucher out shortly after each activity. You will be asked to indicate if you prefer to receive an e-gift card or a physical card that will be sent to you in the mail (postal address required).

| **Risks** |
| --- |

In participating in the panel meetings, there are minimal risks such as:

- Psychological discomfort or distress including emotional responses or unpleasant personal memories due to sharing personal experiences related to cancer prevention behaviours and/or cancer as such or being and speaking in a group setting.
- Concerns or feelings of obligation if you already have a relationship with Cancer Council Queensland and/or the University of Southern Queensland.
- Perceived devaluation of personal worth as a result of group dynamics and the conversations held between and amongst group members. For example, another group member may share their thoughts and opinions on a specific topic and thereby intentionally or unintentionally hurt your feelings. It is also possible that you may feel disadvantaged or feel that your opportunity to contribute is limited where there is one group member that dominates the meeting by not giving others space and/or time to talk.
- Illegal disclosure of your personal information by another group member.

Sometimes thinking about the sorts of issues raised in the group meetings can create some uncomfortable or distressing feelings. If you need to talk to someone about this immediately, please contact:

- Cancer Council (13 11 20): a free confidential support service (Mon–Fri between 9am–5pm)
- Lifeline (13 11 14): a free phone-based, anonymous 24/7 counselling service
- Beyond Blue (1300 224 636): a free confidential 24/7 support service (phone, chat, email)

You may also wish to consider consulting your General Practitioner (GP) for additional support and/or:

- The project team: Larry Myers ([LarryMyers@cancerqld.org.au](mailto:LarryMyers@cancerqld.org.au) or +61 7 3634 5120) or Beatrice Murawski ([BeatriceMurawski@cancerqld.org.au](mailto:BeatriceMurawski@cancerqld.org.au) or +61 7 3634 5251 )
- The University of Southern Queensland Human Research Ethics Committee (Contact details below)

| **Privacy and confidentiality** |
| --- |

All comments and responses are confidential unless required by law.

Please be advised that although the research team will take every precaution to maintain the confidentiality of the data, the nature of group discussions and/or other panel activities prevents the research team from guaranteeing confidentiality. Please respect the privacy of other group members and do not repeat what is discussed during the group meetings with others.

Each panel meeting will be audio and/or video recorded. This allows us to accurately transcribe and summarise all input and contributions made by group members without the risk of missing important details. Recordings and all other participant data will be stored on password-protected servers and accessible only by authorised members of the research team. Transcriptions will be completed by members of the research team listed as investigators on this project and any identifying information (including your name and contact information) will be removed prior to data storage, publication or sharing with other researchers or external collaborators. Your contact details will not be shared under any circumstances; and you will only ever be contacted by the research team and for the purpose of the research outlined in this information sheet.

It is not possible to participate in panel meetings without being audio/video recorded. However, you will be able to withdraw some or all the information you have provided during a panel meeting without having to give reasons. Whilst we cannot delete your data from the actual recordings, your data will not be used for further analysis and reporting if you decide to withdraw it. Your decision to remove your data will in no way or form affect your relationship with CCQ, UniSQ or members of the research team and/or organisation and have no implications for your participation in the rest of the panel meetings planned for this research. You will still be reimbursed for participating in a session, even if your data won't be used for analysis and reporting purposes. Please contact the research team if you have any questions about this.

The same rules apply to the data you provide on the consent form and during the individual think aloud activity and your online survey. Any information that identifies you will be kept separate from these data. All data will be stored securely, as per University of Southern Queensland’s [Research Data and Primary Materials Management Procedure](https://policy.usq.edu.au/documents/151985PL#4.3), on password-protected internal servers managed by Cancer Council Queensland IT Services and/or in locked filing cabinets. Access to any de-identified data collected in this research will be strictly controlled and limited to authorised research personnel. Only investigators named on the application and designated CCQ researchers (for example, statisticians) will have access to the data.

Short plain English language summaries containing group-level results will be emailed to you at the end of the project (e.g., within 6 months after the last panel meeting has taken place). These reports will disclose no identifying information (e.g., name, age), but may include quotes or statements to support our key findings. For example, to show how the needs and preferences you have raised were used to develop the digital platform.

This project is funded by Cancer Council Queensland and conducted by researchers from the Viertel Cancer Research Centre, under leadership of Dr Larry Myers who is affiliated with the University of Southern Queensland.

| **Consent to participate** |
| --- |

You have been invited to enrol as a consumer panel member following your expression of interest. We would like to confirm your agreement to participate in this project prior to scheduling any group meetings. We ask you to send us an email stating the following:

“I have read and understood the participant information statement regarding this project and consent to be part of the study. I have had any questions answered to my satisfaction and I understand that if I have any additional questions, I can contact the research team. I am over 18 years of age. I understand that any data collected may be used in future research activities and that all online panel meetings will be audio/video recorded.”

Your email will be accepted as an indication of your consent to participate in this project.

| **Questions** |
| --- |

Please refer to the Research team contact details at the top of the form to have any questions answered or to request further information about this project.

| **Concerns or complaints** |
| --- |

If you have any concerns or complaints about the ethical conduct of the project, you may contact the University of Southern Queensland, Manager of Research Integrity and Ethics on +61 7 4631 1839 or email [researchintegrity@usq.edu.au](mailto:researchintegrity@usq.edu.au) quoting reference number ETH2023-0359. The Manager of Research Integrity and Ethics is not connected with the research project and can address your concern in an unbiased manner.

**Thank you for taking the time to help with this research project.**

**Please keep this document for your information.**
