# Supplementary material for: Co-design of Lifestyle6, a digital tool targeting multiple health behaviour changes for cancer risk reduction and early detection support
Source: PLoS One. 2026 Apr 16;21(4):e0347311. doi: 10.1371/journal.pone.0347311 (PMC13086309; doi:10.1371/journal.pone.0347311)
Supplement: S5 File — (PDF) [file pone.0347311.s005.pdf]

Privacy & Confidentiality Agreement

As a community representative, it is possible that you will hear, see or be given confidential information as part of your participation. This information may include personal details about panel members, their families or professionals and practitioners. It may also include details about future plans, projects or money matters.

In line with the Australian Privacy Act (1988), during your involvement (and after your involvement ends), all confidential information must be treated in the strictest confidence. You must not remove, destroy, or share any personal information unless the research team at Cancer Council Queensland (CCQ) confirms to you that it is acceptable and lawful to do so and we ask you not to discuss confidential information with anyone outside of the CCQ research team and the group of panel members (this includes family or friends).

Any papers and records containing confidential information must not be copied or left in a way that unauthorised persons can obtain access to them and must be kept safety and securely when not being used.

Your personal information will be kept secure and confidential at all times. CCQ will also ensure that any members of the research team that have not signed a confidentiality agreement as part of their employment or association with this CCQ will be required to sign such an agreement.
