# Supplementary material for: Co-design of Lifestyle6, a digital tool targeting multiple health behaviour changes for cancer risk reduction and early detection support
Source: PLoS One. 2026 Apr 16;21(4):e0347311. doi: 10.1371/journal.pone.0347311 (PMC13086309; doi:10.1371/journal.pone.0347311)
Supplement: S6 File — (DOCX) [file pone.0347311.s006.docx]

**S6 File: Ground rules for participation in online workshops**

**GROUND RULES**

We ask if you can review these ground rules and think about anything else, we should agree on at the beginning of the session.

**Respect**

- There aren’t any right or wrong or silly answers – just ideas, experiences and opinions that are all valuable.

**Confidentiality**

- Any personal experiences that are shared should be kept confidential.

**Preparation**

- Everyone needs to have read any information/references (sent in reasonable time) prior to the meeting.

**Speaking in a group**

- Only one person should talk at a time and contributions must be relevant to the session objectives.

**Inclusion**

- Everyone’s ideas and opinions need to be heard. People who are more confident about speaking should remember to hold back at times and give others a chance.

**Agree to disagree**

- It is important for us to hear all sides of an issue. If people have different views it means we are capturing a variety of opinions and experiences.

**Disclosure**

- We can learn a lot from each other by sharing experiences, but no one should feel obliged to disclose anything they don’t feel comfortable with.

**Stick to the topic**

- As we may discuss issues that are close to people’s hearts and experience, it’s natural that we will all have lots of things to say. As we only have a limited time, we agree to focus on the objective of each session and to park ideas for later discussion.

**Jargon**

- We need to think about the language and acronyms we use to make sure everyone understands. Please ask if anyone uses a term you don’t understand.

**Time keeping**

- We have a lot to get through so it’s important we all come back on time after breaks.

**Mobile phones**

- Everyone should agree to keep their mobile phones and technology switched off or on silent. If you need to use your phone, please do so outside of the room to avoid distracting anyone.
