# Supplementary material for: Co-design of Lifestyle6, a digital tool targeting multiple health behaviour changes for cancer risk reduction and early detection support
Source: PLoS One. 2026 Apr 16;21(4):e0347311. doi: 10.1371/journal.pone.0347311 (PMC13086309; doi:10.1371/journal.pone.0347311)
Supplement: S7 File — (PDF) [file pone.0347311.s007.pdf]

ONLINE SURVEY

COMMUNITY PANEL MEMBER FEEDBACK

Introduction

Over the past months, you have contributed to the development of an innovative digital platform that will be freely accessible to members of the community who seek cancer prevention and screening information and support.

We are interested in your feedback about the workshops and activities you have participated in. This feedback will help us refine and improve any future research activities.

Instructions

The following questionnaire includes several statements and open response items.

Please indicate your level of agreement with each statement and check only one box for each statement. For open response items, please provide a brief but honest answer.

All information you provide will remain confidential.

Thank you very much for your participation.

Community Panel Participation

		Strongly Disagree	Disagree	Neither Disagree nor Agree	Agree	Strongly Agree
1	I had a clear understanding of the purpose of the group workshops.	◯	◯	◯	◯	◯
2	I had a clear understanding of the purpose of the individual ('think aloud') workshop.	◯	◯	◯	◯	◯
3	The supports I needed to participate (e.g., videoconferencing support) were available.	◯	◯	◯	◯	◯
4	I had enough information to contribute to the topics being discussed.	◯	◯	◯	◯	◯

5 What else would you like us to know about how your participation in the project was supported?

____

Community Panel Participation

		Strongly Disagree	Disagree	Neither Disagree nor Agree	Agree	Strongly Agree
6	I was able to express my views freely.	○	○	○	○	○
7	I feel that my views were heard.	○	○	○	○	○
8	A wide range of views on the topic was shared.	○	○	○	○	○
9	The individuals participating in the group meetings represented a broad range of perspectives on the topic.	○	○	○	○	○

10 What else would you like us to know about how you were able to share your views?

____

Community Panel Participation

		Strongly Disagree	Disagree	Neither Disagree nor Agree	Agree	Strongly Agree
11	I think that the group workshops achieved their objectives.	○	○	○	○	○
12	I think that the individual ('think aloud') workshop achieved its objectives.	○	○	○	○	○
13	I am confident the input provided through the group workshops and the individual ('think aloud') workshop will be used by the Cancer Council Queensland research team.	○	○	○	○	○
14	I think the input provided through these activities will make a difference to the work of Cancer Council Queensland researchers.	○	○	○	○	○

15 What else would you like us to know about the influence you think your participation and input will have? __________________________________

Community Panel Participation

		Strongly Disagree	Disagree	Neither Disagree nor Agree	Agree	Strongly Agree
16	As a result of my participation in the project, I am better informed about digital cancer prevention and screening support resources.	○	○	○	○	○
17	Overall, I was satisfied with the group workshops (e.g., group size, structure, duration and frequency of workshops).	○	○	○	○	○
18	Overall, I was satisfied with the individual ('think aloud') workshop (e.g., duration, effort required).	○	○	○	○	○
19	Participating in this project was a good use of my time.	○	○	○	○	○

20 What did you enjoy the most about your involvement in the group workshops?

____

21 What did you enjoy the least about your involvement in the group workshops?

____

22 How could we improve the group workshops next time?

____

23 What did you enjoy the most about the individual ('think aloud') workshop?

____

24 What did you enjoy the least about the individual ('think aloud') workshop?

____

25 How could we improve the individual ('think aloud') workshop?

____

26 What other ideas or thoughts do you have regarding your experience participating in this project?

____
