# Supplementary material for: Co-design of Lifestyle6, a digital tool targeting multiple health behaviour changes for cancer risk reduction and early detection support
Source: PLoS One. 2026 Apr 16;21(4):e0347311. doi: 10.1371/journal.pone.0347311 (PMC13086309; doi:10.1371/journal.pone.0347311)
Supplement: S8 File — (DOCX) [file pone.0347311.s008.docx]

**S8 File. Workshop structure and activities by stage.**

Workshops generally commenced with an Acknowledgement of Country (i.e., a formal recognition of the traditional custodians of the land on which an event is taking place in Australia and to pay respect to all Aboriginal Elders past, present, and emerging), which was followed by an introduction of the facilitators and group members.

**Stage 1 workshops (group-based format)**

As part of the Stage 1 workshops, the scope, timeline, and objectives of both the overall project and the specific stage were introduced, along with a 15-minute presentation on cancer prevalence in Queensland and modifiable cancer risk factors. Group members also completed an icebreaker activity as part of their first workshop to have the opportunity to familiarise themselves with the Teams platform and to get to know each other. The remainder of the Stage 1 workshops was spent viewing an example health research website unrelated to cancer, followed by a group discussion where the facilitator prompted group members to think about what they liked and disliked about the solution and what it would require to be suitable for use in a cancer prevention and early detection context. Group members were directed to an online collaboration tool called Padlet where they could note down their thoughts while browsing the example website.

**Stage 2 workshops (group-based format)**

Subsequent workshops had an identical introduction, but instead of the background presentation, proceeded to give a recap of the preceding workshop, followed by a range of group activities. The stage 2 workshops were made up of three dedicated activities, with the first activity involving the presentation of personas (i.e., fictional characters based on real data from stage 1), each representing a theme related to the frustrations and barriers identified in the first workshop. Panel groups were asked to work together and use this information to craft a succinct problem statement (i.e., point-of-view). Using an empathy map concept, they were asked to think about what their persona would likely say, think, feel or do when searching for cancer risk reduction and/or early detection resources, followed by an activity where the panel groups were asked why that might be and once they had formed a new statement, they were asked again why that might be. This was repeated up to four times until a final problem statement was formed which represented a problem or challenge that can be changed feasibly. These problem statements were then reframed into actionable needs statements, Example prompt (fill the gaps): Persona 1 needs __ to do/be __, because of __.

**Stage 3 workshops (group-based format)**

The needs statement in stage 2 formed the starting point for the Stage 3 activities that focussed on brainstorming solutions on how the problems and needs expressed in previous sessions might be addressed and how this would translate into program contents and/or components of the digital prototype. Example prompts: How might we make information more accessible to the end-user? How might we make information engaging and useful?

**Stage 4 workshops (individual format)**

The Stage 4 workshops were different in that they had a one-on-one format (i.e., one facilitator supporting one panel member per session), were shorter in duration (i.e., 1 hour per panel member) and followed a think aloud protocol. The purpose of using a think aloud protocol was to enable panel members to verbalise their thoughts and decisions when browsing the prototype, provide feedback and suggestions on how the prototype can be improved. It took panel members approximately 30–45 minutes to view, described and comment on each screen. Facilitators remained quiet for most of this period but answered questions and prompted panel members to elaborate further where deeper insights were required.
