# Supplementary material for: Co-design of Lifestyle6, a digital tool targeting multiple health behaviour changes for cancer risk reduction and early detection support
Source: PLoS One. 2026 Apr 16;21(4):e0347311. doi: 10.1371/journal.pone.0347311 (PMC13086309; doi:10.1371/journal.pone.0347311)
Supplement: S9 File — (DOCX) [file pone.0347311.s009.docx]

**S9 File. Linking intervention functions to relevant behaviour change techniques (BCT’s).**

| **Intervention Function** | **Individual BCT’s** |
| --- | --- |
| *Education*  (Increasing knowledge and understanding by informing, explaining, showing and correcting) | - Information about antecedents - Information about health consequences - Information about emotional consequences - Information about other’s approval - Reattribution - Self-monitoring behaviour - Cue signalling reward |
| *Persuasion*  (Changing the way people feel about a behaviour by generating cognitive dissonance and showing how changing behaviour can reduce it) | - Credible source - Information about health consequences - Information about emotional consequences - Salience of consequences - Verbal persuasion about capability - Framing/reframing - Identity associated with changed behaviour - Identification of self as role model - Feedback on behaviour - Social comparison |
| *Incentivisation*  (Changing the attractiveness of a behaviour by creating the expectation of a desired outcome or avoidance of an undesired one) | - Feedback on behaviour - Self-monitoring of behaviour - Reward approximation - Self-reward - Social reward - Incentive - Commitment - Behavioural contract - Discrepancy between current behaviour and goal |
| *Training*  (Increasing psychological or physical skills, or habit strength by explanation, demonstration, practice, feedback and correction) | - Instruction on how to perform a behaviour - Feedback on the behaviour - Self-monitoring of behaviour - Behavioural practice/rehearsal - Habit formation - Graded tasks |
| *Environmental restructuring* (Constraining or promoting behaviour by shaping the physical or social environment) | - Restructuring the physical environment - Restructuring the social environment - Satiation - Exposure - Prompts/cues |
| *Enablement*  (Providing support to improve ability to change in a variety of ways not covered by other intervention functions e.g. through medication, surgery, encouragement, moral support) | - Social support - Goal setting (behaviour and outcome) - Action Planning - Problem solving - Self-monitoring of behaviour - Review of (behavioural and outcome) goals - Behaviour substitution |

*Note*. From “The behavior change technique taxonomy (v1) of 93 hierarchically clustered techniques: building an international consensus for the reporting of behavior change interventions” by Michie et al. (2013) (<https://doi.org/10.1007/s12160-013-9486-6>). Copyright 2013 by The Society of Behavioural Medicine.

External programmes/services that are promoted through Lifestyle 6 may consist of additional BCT’s that are not listed in this overview.
