# Supplementary material for: Co-design of Lifestyle6, a digital tool targeting multiple health behaviour changes for cancer risk reduction and early detection support
Source: PLoS One. 2026 Apr 16;21(4):e0347311. doi: 10.1371/journal.pone.0347311 (PMC13086309; doi:10.1371/journal.pone.0347311)
Supplement: S10 File — (DOCX) [file pone.0347311.s010.docx]

**S10 File. Prototype screenshots**

| 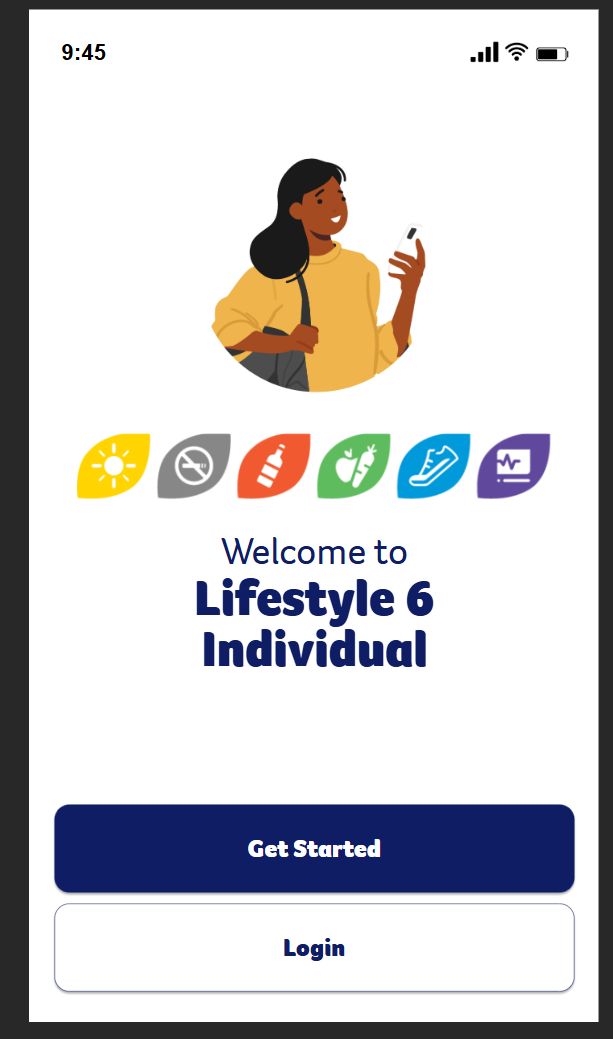 | 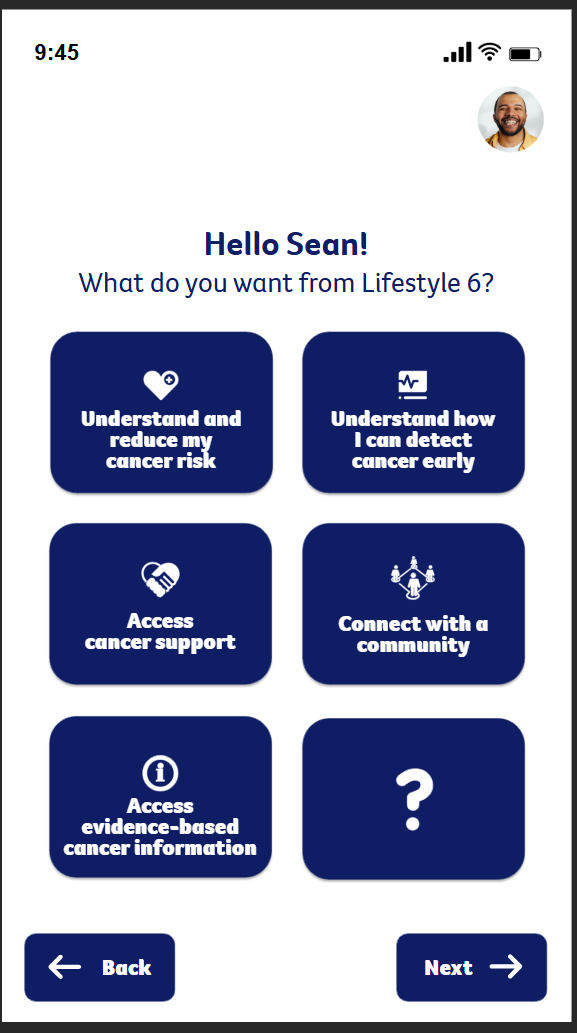 |
| --- | --- |
| Welcome | Preferences |
| 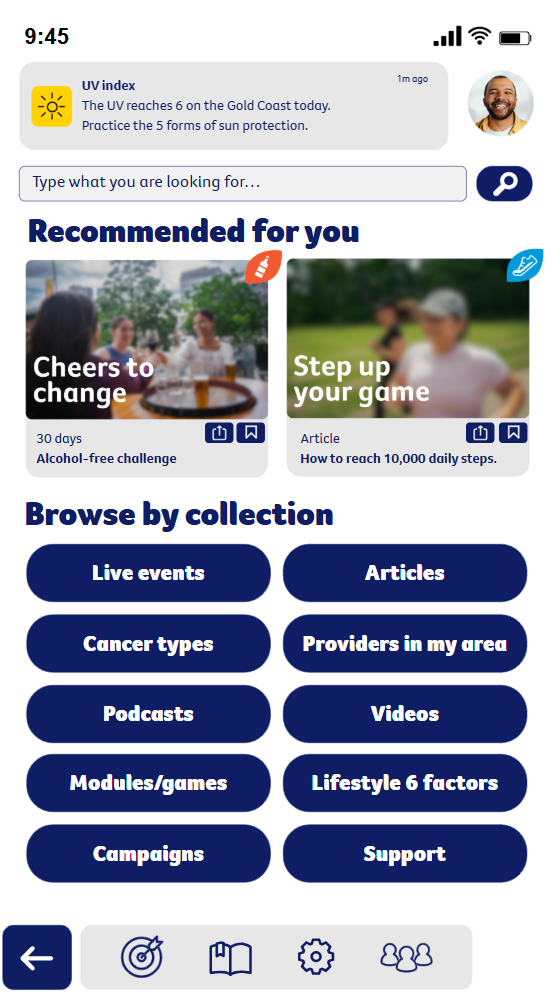 | 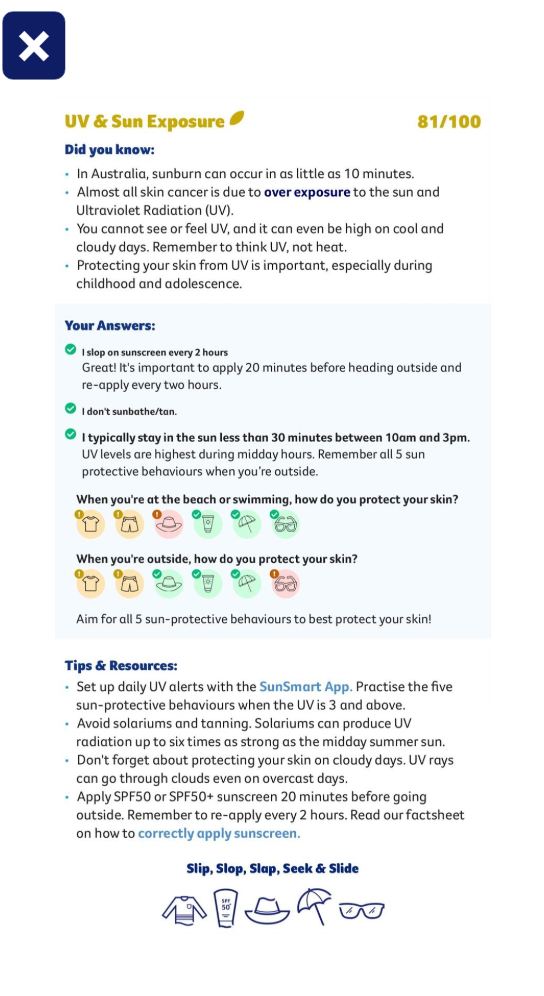 |
| Main dashboard | Sun and UV fact sheet |
| 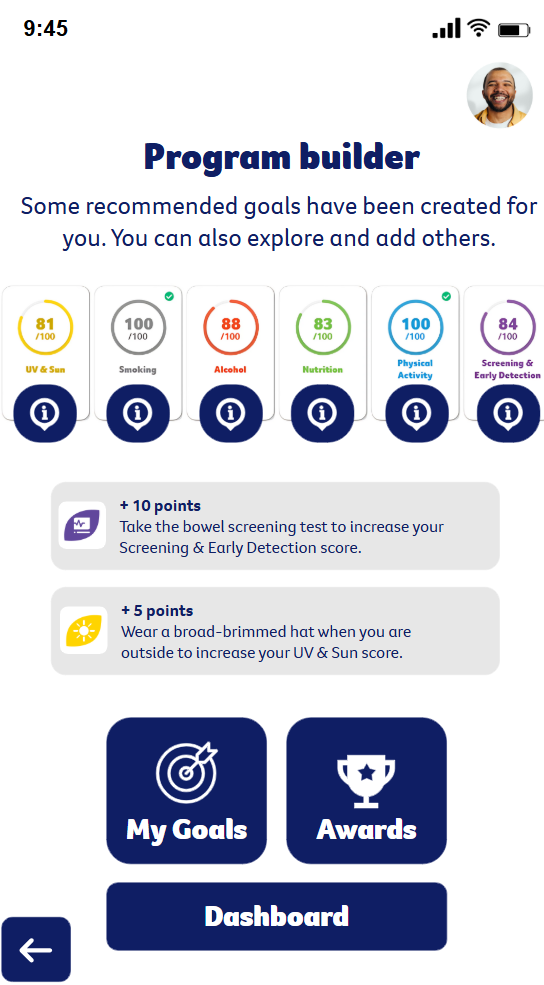 | 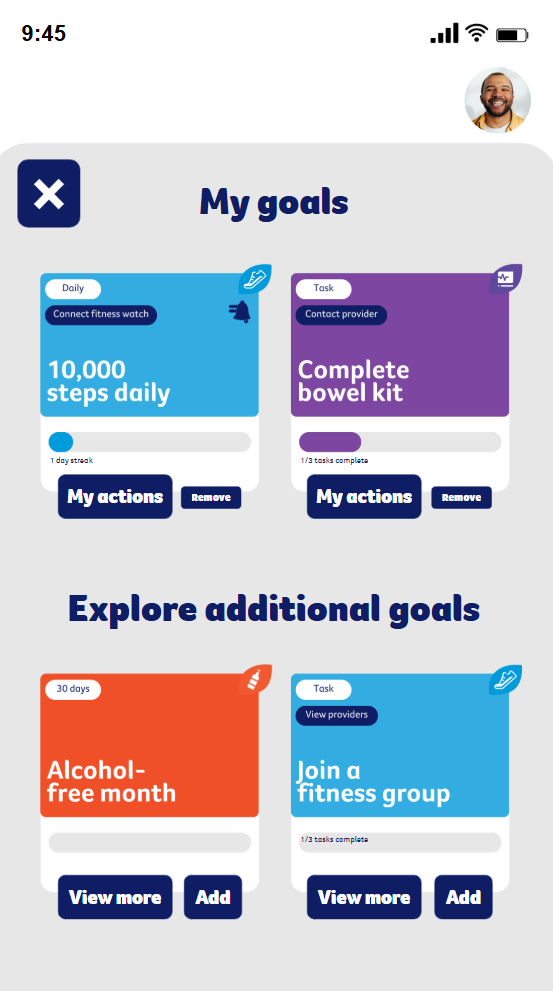 |
| Program builder | My goals |
