# Supplementary material for: Co-design of Lifestyle6, a digital tool targeting multiple health behaviour changes for cancer risk reduction and early detection support
Source: PLoS One. 2026 Apr 16;21(4):e0347311. doi: 10.1371/journal.pone.0347311 (PMC13086309; doi:10.1371/journal.pone.0347311)
Supplement: S11 File — (DOCX) [file pone.0347311.s011.docx]

**S11 File. Prototype feedback from community panel members.**

| **Physical Capability**  Features that enable users to physically interact with the app | - Ensure fonts & buttons are easy to read (especially for older users) - Ensure text and buttons are large enough for accessibility - Provide smooth transition between pages - Make graphics and icons clearer & more engaging |
| --- | --- |
| **Psychological Capability**  Features that support knowledge, understanding, and cognitive processing | - Avoid information overload, break content into digestible sections - Provide tooltips or help buttons for unclear terms - Ensure cancer information is evidence-based & easy to access - Offer clearer explanations of risk factors & how to address them - Provide clear, concise health recommendations instead of excessive text - Include information on choosing effective sunscreen & proper usage - Ensure risk calculator is personalised & results feel tailored |
| **Physical Opportunity**  Environmental features and resources that make the behaviour possible | - Simple landing page & onboarding process - Ensure consistency in design & layout - Smooth search functionality - Improve navigation (clear home button, back button, or main menu access) - Allow users to browse by categories - Provide multiple content formats (articles, podcasts, videos) - Allow users to see UV index information in real-time for sun protection - Enable digital filters to help users see only relevant content - Allow bookmarking of articles/resources for later reference |
| **Social Opportunity**  Cultural and social factors that support the behaviour | - Include real-life testimonials & personal stories for relatability - Improve community group visibility & accessibility - Ensure community support links work & include relevant groups - Clarify how users can connect with others - Provide options to find local support services - Include financial and mental health support resources - Enable social sharing or challenges |
| **Reflective Motivation**  Conscious planning and evaluation that drives behaviour | - Enable step-by-step goal setting with progress tracking - Include personal goal-setting options - Make progress tracking clearer with a score system - Ensure a clear progress-tracking system with visible scores - Provide practical feedback instead of just badges - Include warnings or real-life consequences for not taking preventive actions - Include user profiles with history tracking & saved preferences - Provide a personalised dashboard with tailored recommendations |
| **Automatic Motivation**  Emotional reactions, desires, and habits | - Use personalised greetings & onboarding questions - Allow users to toggle features on/off (badges, notifications, etc.) - Provide customisable screening reminders - Provide an option to customise notifications & reminders - Make awards/badges optional - Provide tangible rewards or incentives for goal completion - Enable notifications or updates for new community groups or discussions - Ensure app adjusts recommendations based on user input |
|  | |
