# Supplementary material for: Co-design of Lifestyle6, a digital tool targeting multiple health behaviour changes for cancer risk reduction and early detection support
Source: PLoS One. 2026 Apr 16;21(4):e0347311. doi: 10.1371/journal.pone.0347311 (PMC13086309; doi:10.1371/journal.pone.0347311)
Supplement: Attachment — Submitted filename: Response to reviewers_22122025.docx [file pone.0347311.s013.docx]

**Response to Reviewers – 4/12/2025**

Thank you to the reviewers for their time reviewing our submission and for their valuable feedback. We feel their insights have enhanced the quality of this manuscript. Please find below a point-by-point description of how the suggestions have been implemented. The ‘section’ column corresponds with the marked-up version of the manuscript, which includes numbered comments indicating where changes have been implemented.

**Journal requirements**

| **Comment** | **Response** | **Section** |
| --- | --- | --- |
| Please ensure that your manuscript meets PLOS ONE's style requirements, including those for file naming. | The following files have been amended according to PLOS ONE’s style requirements:   - ‘Revised Manuscript with Track Changes’ includes the manuscript with tracked changes. - ‘Manuscript’ includes an unmarked version of the paper without tracked changes. | n/a |
| Please state what role the funders took in the study.  If the funders had no role, please state: "The funders had no role in study design, data collection and analysis, decision to publish, or preparation of the manuscript."  If this statement is not correct you must amend it as needed.  Please include this amended Role of Funder statement in your cover letter; we will change the online submission form on your behalf. | Apologies for this oversight. We have amended the funding statement to clarify that the funders had no role in study design, data collection and analysis, decision to publish, or preparation of the manuscript on the cover letter for this submission. | n/a |
| a) If there are ethical or legal restrictions on sharing a de-identified data set, please explain them in detail (e.g., data contain potentially identifying or sensitive patient information, data are owned by a third-party organization, etc.) and who has imposed them (e.g., a Research Ethics Committee or Institutional Review Board, etc.). Please also provide contact information for a data access committee, ethics committee, or other institutional body to which data requests may be sent. | The data that comprise the findings of this study are held securely on password protected servers and are only currently accessible by members of the research team granted permission from the University of Southern Queensland Human Research Ethics Committee (ref. ETH2023-0359). The data are unable to be uploaded to a publicly available server due to the potentially identifying nature of the information; however, an aggregated summary of the data may be requested by other parties (e.g., researchers) upon reasonable request to the corresponding author. The merit of each request will be individually evaluated by members of the research team before provision of de-identified interview materials. | n/a |
| Please upload a new copy of Figure 2, as the detail is not clear. | We apologise for this oversight. Due to concerns related to copyrighted images used in Figure 2, we have decided that it is best to remove this figure from the manuscript. Instead, we now provide additional text in the main manuscript to describe the purpose and function of Figure 2 (a virtual storyboard developed to capture key insights from panel members, used to inform the intervention prototype) (see pg 21, lines 331-336):  *“To develop the visual representation of the prototype, HG initially created a draft storyboard in* [*Miro*](https://miro.com/index/) *incorporating the community panel themes.* ***The storyboard provided a central representation of the key insights shared by panel members their needs and preferences for the functionality of the prototype. It drew on user-suggested examples from existing apps developed to support health-related behaviour change.”*** | n/a |
| We note that Figures 2 and S4 in your submission contain copyrighted images. All PLOS content is published under the Creative Commons Attribution License (CC BY 4.0), which means that the manuscript, images, and Supporting Information files will be freely available online, and any third party is permitted to access, download, copy, distribute, and use these materials in any way, even commercially, with proper attribution. For more information, see our copyright guidelines: http://journals.plos.org/plosone/s/licenses-and-copyright.    We require you to either present written permission from the copyright holder to publish these figures specifically under the CC BY 4.0 license, or remove the figures from your submission:    a. You may seek permission from the original copyright holder of Figures 2 and S4 to publish the content specifically under the CC BY 4.0 license. | We apologise for this oversight.  As noted above, due to the concerns related to copyright raised herein, we have decided to remove Figure 2 from the manuscript. We have added additional text within the main manuscript to explain to readers the purpose of the storyboard and its use in the development of the intervention prototype to ensure there is no missing context.  Please note that S4 File (now relabelled as S7 File) does not include any images. We acknowledge that the Behaviour Change Technique Taxonomy (Michie et al. 2013) was not clearly cited in this document, however, and we have amended this as follows:  *Note. From “The behavior change technique taxonomy (v1) of 93 hierarchically clustered techniques: building an international consensus for the reporting of behavior change interventions” by Michie et al. (2013) (*[*https://doi.org/10.1007/s12160-013-9486-6*](https://doi.org/10.1007/s12160-013-9486-6)*). Copyright 2013 by The Society of Behavioural Medicine.*  Please let us know if you require any further action to resolve concerns related to copyright. | n/a |
| We note that this data set consists of interview transcripts. Can you please confirm that all participants gave consent for interview transcript to be published?  If they DID provide consent for these transcripts to be published, please also confirm that the transcripts do not contain any potentially identifying information (or let us know if the participants consented to having their personal details published and made publicly available). We consider the following details to be identifying information:  - Names, nicknames, and initials  - Age more specific than round numbers  - GPS coordinates, physical addresses, IP addresses, email addresses  - Information in small sample sizes (e.g. 40 students from X class in X year at X university)  - Specific dates (e.g. visit dates, interview dates)  - ID numbers | We can confirm that participants gave consent for the interview transcripts to be published, of which de-identified excerpts are presented in this manuscript.  We have amended the manuscript to clarify this as follows (see pg 7 lines 163-165):  *“Selected individuals were notified via email and asked to provide informed consent to participate* ***(including consent to publish de-identified excerpts of the interview transcripts)*** *via reply email.”*  We can confirm that excerpts of the transcripts included in the manuscript do not include any of the identifying details listed herein. | n/a |

**Reviewer 1**

| **Comment** | **Response** | **Section** |
| --- | --- | --- |
| The adoption of videoconferencing for engagement with panel members is likely to have aided recruitment and engagement. Was the adoption of this engagement strategy motivated by a review of the success of online engagement in other studies or was this for convenience? A comment on this choice could be added. | Thank you for this suggestion. We have clarified the rationale for the use of online interview panels as part of this study (see pg 8-9, lines 195-197):  *“****Given the established benefits of online interview methods, including greater convenience, participant reach, and retention (46),*** *all workshops were hosted on Microsoft Teams, recorded, and auto-transcribed.”* | 1.1 |
| Was there any PPI involvement in the design of the engagement process? | There was no public and patient involvement in the design of the engagement process. We opted to use videoconferencing for data collection in this study based on our prior experience – usually, we offer participants the opportunity to participate in person or online, and we have noted a strong preference for online participation given its convenience. We also thought that videoconferencing was most appropriate given our aim to recruit people across Queensland, including those in rural and remote areas. | n/a |
| How the participants were allocated to the initial 5 groups could do with further explanation, particularly how homogeneity was defined. Was it intended that Group 1 would all be IT professionals? Same for the group comprised of community workers. Why did each group consist of females or males only? Explain why homogeneity might be appropriate. | We now provide some further detail and rationale as to why this homogeneity was not disrupted and how it may have influenced our findings in the Results section (pg 13-14, lines 274-280):  *“At the outset, two of the groups consisted of all male members, three groups consisted of all female members and a wide age range was represented in all five groups.* ***The homogeneity observed between gender (all groups) and occupation (groups 1 and 3) was unintended but the research team did not intervene. Evidence suggests that while heterogenous focus groups facilitate new idea generation, homogeneity can drive connection, honesty, and openness (49,50), both of which are relevant processes in facilitating effective focus groups for this purpose.****”* | 1.2 |
| Timelines for the process should be explained. How much time elapsed between workshops? | Thank you. We have now edited the Results section (see pg 13 lines 264-268) to clarify when each workshop was held throughout 2024:  *“Thirteen group-based and 17 individual workshops were held. Of the 30 consenting individuals, 22 were available to attend a workshop in Stage 1* ***(held in January to February 2024)****, 21 attended in Stage 2* ***(held in April to May 2024)****, 13 in Stage 3* ***(held in August to September 2024)*** *and 17 in Stage 4* ***(held in November 2024)****.”* | 1.3 |
| Participants and recruitment: The limitations section notes that greater representation from low socioeconomic backgrounds, rural and remote communities, and other priority populations would have further enhanced the breadth of insights gained. The online format could have helped with this. Other studies engaged with representative organizations/NGOs in order to reach these seldom heard voices. What consideration was given to reaching these populations? | Thanks for this question. Our recruitment strategy involved online advertising via the official social media profile of a nonprofit cancer support organisation, with a specific attempt to target diverse groups in Australia. Unfortunately, despite our efforts, we were unable to ascertain as much representation from all key priority groups as we had hoped, hence its acknowledgment as a limitation.  In response to this feedback, we now provide some further detail of our attempts to reach these populations in the limitations section (see pg 27, lines 480-486):  *“****Furthermore, despite our efforts to target diverse communities throughout our social media advertising campaign, people from*** *low socioeconomic backgrounds, rural and remote communities, and* ***Aboriginal and Torres Strait Islander populations were still under-represented.*** ***Future research should employ diverse recruitment strategies (such as collaboration with key community organisations) to more comprehensively reach these communities*** *and enhance the breadth of insights gained.”* | 1.4 |
| Additional information could be added to the Supplementary Files: • the participant information statement, the privacy and confidentiality agreement and the ground rules for participation in the online workshops. • The online survey completed by participants. | Thank you. A full list of Supplementary Files is provided on pg 35, including:  S3 File. Expression of interest form  S4 File. Participation information sheet  S5 File. Privacy and confidentiality agreement  S7 File. Online feedback survey | 1.5 |
| On completion of the engagement, were the panel members given an opportunity to provide feedback on the engagement process? | Apologies for this oversight. We have amended our Methods section to clarify that upon completion of all phases of the focus groups, participants were invited to comment on the overall engagement process using the Public and Patient Engagement Evaluation Tool (PPEET) version 2.0 (see pg 8 lines 190-1193):  *“****Upon completion of all focus groups, participants were invited to complete the Public and Patient Engagement Evaluation Tool (PPEET) 2.0 (46,47) to provide feedback on their experience throughout the engagement process****.”* | 1.6 |
| Figure 2 – I struggled to read this image | We apologise for this oversight. Due to feedback from peer reviewers and the journal, we have opted to remove Figure 2 from this publication. We now provide additional explanation within the main manuscript to describe the purpose and function of the storyboard in the development of the prototype (see pg 21, lines 331-336):  *“To develop the visual representation of the prototype, HG initially created a draft storyboard in* [*Miro*](https://miro.com/index/) *incorporating the community panel themes.* ***The storyboard provided a central representation of the key insights shared by panel members their needs and preferences for the functionality of the prototype. It drew on user-suggested examples from existing apps developed to support health-related behaviour change.”*** | 1.7 |

**Reviewer 2**

| **Comment** | **Response** | **Section** |
| --- | --- | --- |
| Line 53: I suggest clarifying that it’s solid tumour cancers for prevention via healthy lifestyle. | Thank you, we have implemented this suggestion as follows (see pg 3, lines 55-57):  *“****In fact,*** *research indicates that 30–50% of* ***cancers*** ***(particularly solid tumours)*** *could be prevented through the adoption of healthy lifestyle behaviours [2–4].”* | 2.1 |
| Lines 57-58: Please carefully re-write this sentence to make evident certain cancer diseases (a small portion) have evidence-based established guidelines for early detection cancer screening. This could be a supporting reason for why it is critical to consider interventions that can address modifiable behaviors cancer risk factors due to limitations with cancer screening for other cancers. | Thank you for this insight. We have incorporated this recommendation to the introduction as follows (see pg 3, lines 60-66):  *“****In addition, among cancers which can be reliably screened for (e.g., breast, bowel, and cervical),*** *screening programs can significantly enhance early detection, reducing mortality rates [7].* ***However, such screening programs do not exist for most cancers, and are typically only available for subsets of the population identified to be at elevated risk (8). Therefore, it is critical to consider lifestyle interventions to reduce cancer risk which may benefit the population as a whole.”*** | 2.2 |
| If you can cite research knowledge relevant to populations in Australia or high-income countries (as a whole), briefly highlight existing evidence related to modifiable cancer risk factors. For example, evidence similar to the paper written by Islami et al. (2024) Proportion and number of cancer cases and deaths attributable to potentially modifiable risk factors in the United States, 2019. CA: A Cancer Journal for Clinicians. | We have incorporated this recommendation and provided some further context to the introduction to describe modifiable cancer risk factors (see pg 3, lines 53-55):  *“****While cancers often arise from immutable factors such as DNA damage and genetic risk factors, a host of lifestyle factors such as tobacco and alcohol use, physical inactivity, and dietary behaviours are also associated with elevated cancer risk (9)****. In fact, research indicates that…”* | 2.3 |
| Lines 132 – 134: There is mention of “panel members.” Were all participants a panel member? Re-word to make it clear that all or some participants’ role was to be engaged as panel member. | Thanks for raising this. We have made edits throughout the manuscript to clarify that all participants were considered community panel members (for example, see pg 7, 155-156):  *“Participants* ***(herein referred to as community panel members)*** *were purposively recruited…”*  Pg 8, lines 190-191: *“Upon completion of all focus groups,* ***panel members*** *were invited to…”* | 2.4 |
| Line 151: Make it clear that the individual workshops or all the workshops were online. | We have incorporated this recommendation. See pg 8, line 175  ***“All workshops were held online”.*** | 2.5 |
| Regarding implications for practice, have you considered the feasibility of maintaining an educational/information dissemination product that is accurate, up-to-date, clearly evidence-based, and engaging for the user? How would the users be encouraged to communicate with GP (or other medical professional) to discuss their unique health situation? | Thank you for this insight. We agree that consideration of how a “lifespan” application can maintain individual relevance and facilitate connection with medical professionals would enhance the quality of this manuscript. We have implemented these suggestions as follows (see pg 25, lines 440-449):  ***“Findings from this study highlight the use of tailored content to suit the individuals’ needs, preferences, and stage of behavioural “readiness” for change as core user priorities. However, this platform is intended to offer continual (rather than acute) support, and therefore a key responsibility of this platform will be to ensure that users are aware of the constraints in knowledge and advice that can be provided in the absence of a personal medical professional. As such, it will be critical to ensure that effective offboarding processes exist, connecting users with healthcare providers for individualised medical support. For example, partnership with community organisations may facilitate the development of self-referral pathways for individuals identified as “high risk” or in need of additional behaviour change support (such as those with high alcohol intake).”*** | 2.6 |
| Strengths and limitations: The web-based participation is convenient and practical since it allows for human subjects to participate from their personnel space. Although have you considered that the web-based participation requirement may have limited certain types of participants from being a valuable informant? Do you think your data reflects well the younger adult population perspectives and experiences (18 – 30 years old)? Future direction could involve in-person study participation and improve recruitment of young adult populations for the next phases of testing the product. | Thanks for this feedback. We agree that our decision to facilitate the focus groups online may have resulted in some level of selection bias, which we now discuss in the Limitations section of the manuscript (see pg 26-27, lines 472-480):  *“The high proportion of panel members with personal cancer experiences or cancer within their immediate social networks* ***may have contributed*** *to elevated subject matter interest and heightened risk perceptions, that may not be typical of the broader population.* ***Similarly, our decision to conduct the focus groups online may have enhanced our reach of participants outside of metropolitan areas, but it may also have resulted in selection bias, such that our sample may have exhibited higher levels of technological literacy than the general population.*** ***Offering in-person focus groups may combat this issue****.”* | 2.7 |

**Reviewer 3**

| **Comment** | **Response** | **Section** |
| --- | --- | --- |
| My overall feedback is that when I read the paper, I remained confused about what was being developed and the process of development. It wasn’t until I saw the supplements showing the interface that it started to make sense to me, and the supplement that described the workshop structure and activities by stage, and the supplement with the Prototype screenshots. I think my feedback is that the manuscript needs a little more structure, and parallel writing to help the reader understand what is being done and why at each step. Naming or numbering each step consistently might help, or perhaps subheadings would be useful. | Thank you for this valuable feedback.  In response, we have made several amendments throughout the manuscript to create consistency and clarity in the flow. As we describe in response to your below comment, this work was informed by the principles of Design Thinking. As such, we now use consistent language throughout the Methods and Results sections to highlight which of the four Design Thinking stages are being described, and create an overarching narrative structure to our work. For example:  Pg 5-6, lines 120-140:  *“This qualitative study was informed by the Learning Health Systems Framework [41] and the Behaviour Change Wheel [42]. The stage-wise community consultation process detailed here, followed established Design Thinking principles (35)]. Design Thinking is a powerful approach to human-centered digital design that* ***offers a multi-stage method for the iterative design and testing of user-informed solutions*** *(41). As such, it suits the complex challenges associated with cancer risk reduction and early detection.* ***The stages of Design Thinking include: Empathise (aiming to understand the issue as experienced by those affected), Define (in which users generate guiding problem statements to anchor the design process), Ideate (in which users generate radical design alternatives), and Prototype (in which user input is synthesised into a trial version of the final product or system presented to users for feedback)*** *(41).* ***Throughout these stages, the Behaviour Change Wheel*** *was integrated to facilitate the systematic translation of qualitative insights into evidence-based,* ***actionable suggestions to inform development of a program capable of eliciting lifestyle behaviour change*** *(44). This manuscript follows the consolidated criteria for reporting qualitative research (COREQ) (S1 File) (45). The study received ethical approval from the University of Southern Queensland Human Research Ethics Committee (Reference #: ETH2023-0359).”*  Pg 8, lines 173-175:  “The design process used in this study encompassed three rounds of group-based online workshops (i.e., **design stages *Empathise, Define,* and *Ideate***), and one round of individual workshops (i.e., **design stage *Prototype***).”  Pg 8, lines 180-183:  “The group-based workshop activities completed in the first three stages generally encouraged divergent thinking without any major design limits imposed, **in alignment with Design Thinking Principles (35).** In contrast, the **Stage 4 (*Prototype)* workshops** were conducted in one-on-one format and took a convergent approach…”  Pg 8, lines 187-188:  “Further details on the format, duration, activities, data collection and analyses **by each Design Thinking stage** is provided in Table 1.”  Furthermore, the subheadings in the Results have been revised to explicitly reflect the stages of Design Thinking. For example:  Pg 15, line 288:  “Summary of panel perspectives: ***Empathise* and *Define***” | 3.1 |
| My second high level feedback is about the interdisciplinary nature of this paper, which is a strength, but the manuscript would be improved by more clearly defining design-thinking and user experience type constructs mre clearly from the beginning of the manuscript. Overall, there was a lot of describing of “behavior change theories”, largely drawn from health promotion, social psych, etc. type fields of behavioral and social sciences without an equivalent set of terms or elements drawn from design fields. This is a missed opportunity. | Thank you for this insight. We agree that further emphasis on Design Thinking and its related principles would enhance the quality and clarity of this work. We now include a more thorough description of Design Thinking and the four key phases of this process which informed the basis of our methodology in the Introduction (see pg 4-5, lines 98-105):  “The use of co-design approaches **such as Design Thinking in intervention development** **have emerged in attempt to combat this issue** (35). **Design Thinking aims to** engage consumers in the conceptualisation, development and refinement of interventions, ensuring that the end product is representative of the target population and their lived experiences[34,35]. This, in turn, may increase the likelihood of successful programme uptake and sufficient engagement (38)].”  We also provide additional description of these phases in the Methods section (as described above) to highlight the four key phases.  We also feel that the edits made to the manuscript in response to your comment above (3.1) aid the reader in understanding how Design Thinking is the common thread which informs all aspects of this manuscript. We have made amendments throughout the Methods and Results sections to consistently highlight which stage of Design Thinking the information pertains to. | 3.2 |
| Line 188: Describe “padlet and chat posts” . As a reader I don’t know what these are. | Thanks for this suggestion. Upon reflection, we feel it was necessary to introduce the online platform, Padlet, earlier in the manuscript, rather than in the results. As such, we have provided a description of what Padlet is and its functionality on pg 8, lines 188-190:  *“****During the workshops, the digital canvas platform, Padlet, was used (49). This allowed panel members to collaboratively brainstorm, sharing ideas and links to other media (such as Word documents) in real time.****”* | 3.3 |
| 191- Define COREQ guidelines and at least spell out. | This recommendation has been incorporated. See pg 6, lines 136-138  *“This manuscript follows the consolidated criteria for reporting qualitative research (COREQ) (see S1 File) (46), a 32-item checklist describing best practice reporting of the methodology, design, and analysis of qualitative interview and focus group studies.”* | 3.4 |
| 192- Clarify who and how “appraise the standard of evidence” was done. This seems like a very important step and there’s not enough details for me to have confidence in this process. | We apologise for this oversight. In retrospect, this sentence is unclear and does not clearly communicate our methodology. We have rephrased the meaning of this sentence (see pg 11, lines 224-226):  *“****Transcripts were reviewed for clarity by interview facilitators with disagreements in perceived meaning resolved through discussion****.”* | 3.5 |
| 201- personas and empathy maps need to be explained somewhere for those who aren’t experts in this topic. I wonder if the design thinking concepts like this could be added to one of the tables in the supplement that lists all the “behavior change theories” that are elements of theories. | Thanks for this feedback. We have added explanations of personas and empathy maps within the manuscript. See pg 12, lines 236-248.  *“****Personas and empathy maps are design thinking techniques used to explore the perspectives and experiences of consumers, ensuring that the end product meaningfully addresses their needs. . Personas involve the development of a series of fictional product users based on interview data, and are intended to anchor the development process around an understanding of who the product is designed for (55). An empathy map is a collaborative tool that enables consumers to identify what product users “say, think, do, and feel”, providing deeper insight into the needs and expectations of the intended user base(55). Together, these tools aim to solidify consumer involvement in the design process to improve user experience of the end product.****”* | 3.6 |
| Grammatical confusion in table 3, last row under theme 1 sentence starting “amongst those eligible…” | Apologies for this error. This has been corrected (see Table 3):  *“Amongst those eligible...concerns were shared about specific barriers to access (e.g., lack of local services, limited relevance/trust****) encountered*** *by those living in rural…”* | 3.7 |
| Line 257- wat is “full settings control”? | This has been defined on pg.17 lines 312-314  *“****Full settings control is a UX concept where users can have visibility, flexibility and mastery over their settings (59), allowing them to tailor the interface (e.g., content) to improve their satisfaction of the experience (60)****”.* | 3.8 |
| Line 256-6: The phrase “This step was completed to facilitate reporting” confuses me. And how is this strong rationale for doing this work? What reporting is being referred to here, and what does that have to do with intervention design and development? | Apologies for this oversight. This sentence was meant to convey that the mapping of recommendations from the Prototype phase to the Behaviour Change Wheel was done for internal reporting purposes. We acknowledge that this was unclear and we have revised this sentence accordingly (see pg 17, lines 322-323):  *“…and the corresponding support strategies of the Behaviour Changes Wheel (44)* ***(see file S4).This visual representation confirmed*** *a balanced distribution of intervention functions across all domains of the Behaviour Change Wheel.”* | 3.9 |
| Line 300- This was confusing to me saying things “can be used” made me wonder- is this how the prototype currently works, or a future adaptation that is possible given the prototype design. | Thank you for raising this. We have revised the tense throughout the description of the prototype to clarify that the functionality described was indeed how the application – at the prototype stage – functioned (see pg 21-22, lines 349-378):  *“The prototype (example screenshots shown in S5 File) was designed as a mobile-friendly application (i.e., for installation on smartphones and tablets) as this was favoured over a standard website by most panel members. To ensure that personal accounts and all user data are kept private, users* ***were*** *required to sign up with their email on first use; after which they* ***were able*** *to log in and out of their account at their convenience.*  *After registering an account, users* ***were provided*** *access to the start page of the programme, which asks them what they would like to get out of Lifestyle 6 with multiple priority areas available. These* ***included****: (1) understand and reduce my cancer risk, (2) understand how I can detect cancer early, (3) access cancer support, (4) connect with a community and (5) access evidence-based cancer information. The dashboard* ***housed*** *evidence-based information (e.g., facts sheets, research articles, videos, podcasts) and a range of support options (e.g., connect with other users by joining a community group, contact helpline, etc.), both of which align with the information provided by users and their selection of priority areas. Pre-filtered content* ***was designed to be*** *amended in the settings to further match individual preferences. Resources of interest* ***were also able to*** *be saved to a personal library for quick access.*  *Users* ***are*** *given the option to either accept the system-generated programme or amend it manually based on their preferences. They* ***were also able to*** *review and gradually adjust their goals in the programme builder section, define the actions they wish to take and/or join existing initiatives or challenges that align with their goal/s. The use of graded tasks and progress towards goals* ***was*** *instantly rewarded with encouraging feedback, whereas goal achievement was rewarded with respective badges. Within the goal setting section, users* ***were able to*** *view lists of current or future events and existing programmes and organisations that promote a healthier lifestyle, such as "10,000 steps" or "Dry July". However, participation in those programmes* ***was*** *entirely optional. Social comparison* ***was facilitated*** *through features that allow users to share their achievements on social media and across in-app community groups available in the prototype. The individual prototype testing workshops were used to seek user feedback (S11 File).”* | 3.10 |
| Fig 2 confusing especially part in top right with game of Life and random images. | We apologise for this oversight. Due to feedback from peer reviewers and the journal, we have opted to remove Figure 2 from this publication. We now provide additional explanation within the main manuscript to describe the purpose and function of the storyboard in the development of the prototype (see pg 21, lines 331-336):  *“To develop the visual representation of the prototype, HG initially created a draft storyboard in* [*Miro*](https://miro.com/index/) *incorporating the community panel themes.* ***The storyboard provided a central representation of the key insights shared by panel members their needs and preferences for the functionality of the prototype. It drew on user-suggested examples from existing apps developed to support health-related behaviour change.”*** | 3.11 |
| Minor point- most of the supplements are mislabeled in the online system file name, making it a little confusing. | Apologies for this oversight. We have amended the labelling of the supplementary files and confirmed that they are correct. | n/a |
| Another minor point. Several times it is mentioned that intervention components are matched behavioral change theories. This is a little bit of a stretch because few theories are listed, it’s specific theoretically-driven constructs that are being discussed. | Thank you for raising this.  We have reviewed the manuscript and can confirm that we refer to behaviour change theories only once explicitly. We agree that this is not as accurate as describing these as “behaviour change constructs” and thus we have modified the following sentence (see pg 12-13, line 250-251):  *“To support prototype design, we applied…based on established behaviour change* ***constructs****.”* | 3.12 |
